# Establishment of Positional Signals in Megaspore Mother Cell Specification: An Updated Review

**DOI:** 10.3390/plants15203184

**Published:** 2026-10-17

**Authors:** Ling Sun, Tingyu Liu, Yufei Chen, Hanyang Cai, Yuan Qin

**Affiliations:** Haixia Institute of Science and Technology, School of Future Technology, College of Life Sciences, Fujian Agriculture and Forestry University, Fuzhou 350002, China; sunling1998@163.com (L.S.); 15904444973@163.com (T.L.); huihui9233@163.com (Y.C.)

**Keywords:** MMC specification, positional signals, positional competence, phytohormone gradients, mobile signals, cytoskeleton components

## Abstract

The megaspore mother cell (MMC), the initial cell of the female germline in flowering plants, differentiates from a distal subepidermal somatic cell in the ovule. Positional signals guide this process, yet the mechanisms by which multiple signals integrate to specify a single MMC remain poorly understood. Here, we summarize recent advances in MMC specification, focusing on the interplay between positional signals and positional competence. Positional signals include phytohormone gradients, mobile cues, cell wall-derived physical boundaries, and mechanical signals. Positional competence is the ability of a cell to interpret these signals. We propose an integrated model in which chemical gradients define the potential reproductive region, mobile signals establish inhibitory fields, the cell wall gates signal movement, and the cytoskeleton coordinates signal delivery, division-plane patterning, and mechanical transduction. This review provides a conceptual basis for understanding how a single somatic cell is selected to acquire MMC identity and highlights potential targets for further research and crop improvement.

## 1. Introduction

**Figure 1:**
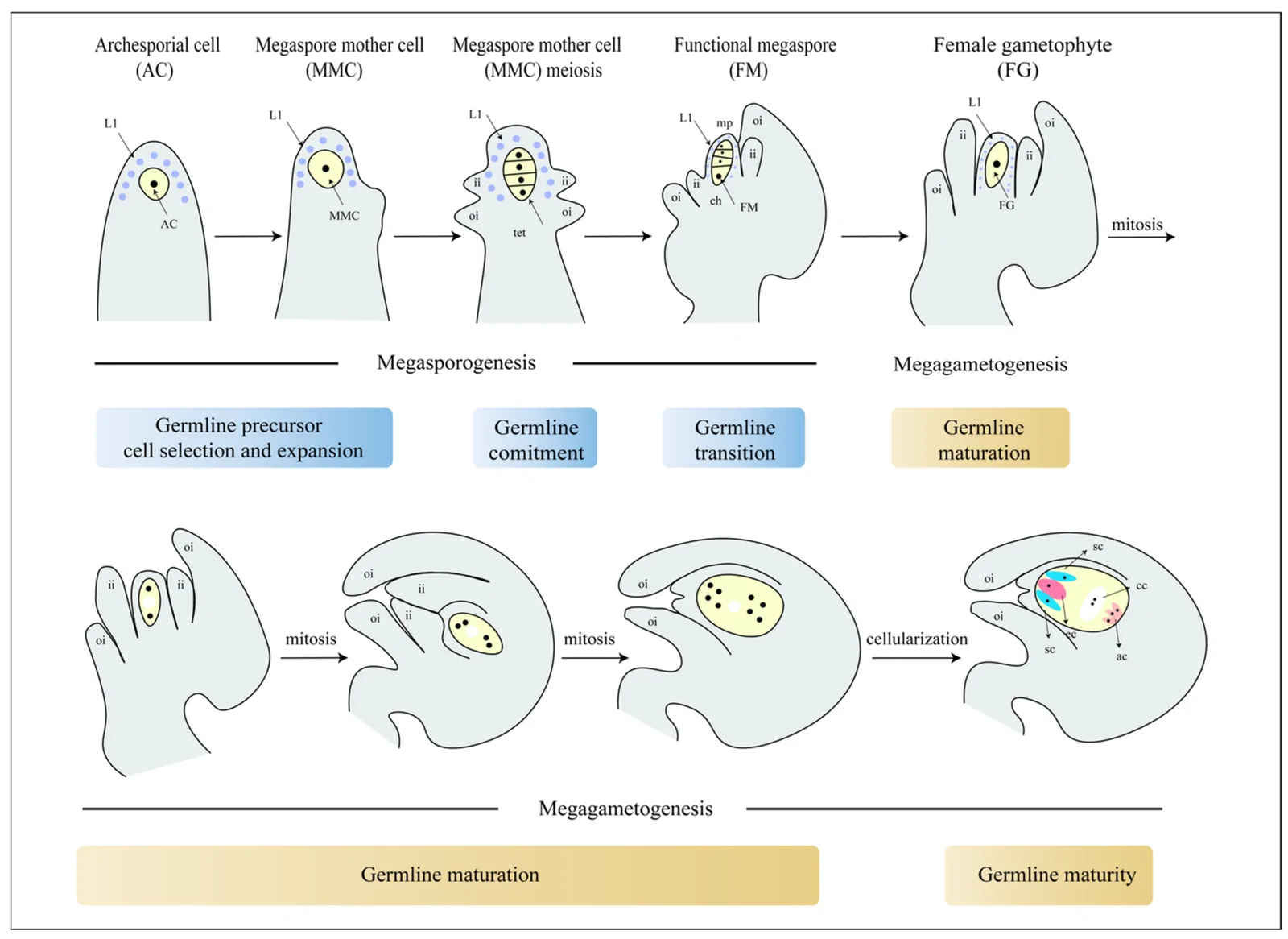
Development of the female germline in *Arabidopsis thaliana*. Female gametophyte development includes megasporogenesis and megagametogenesis. This process initiates from the subepidermal layer (L2) nucellar cell at the distal end of the ovule primordium, which differentiates into an archesporial cell (AC), and then differentiates into the megaspore mother cell (MMC). The MMC undergoes meiosis to form four haploid megaspores. Among them, three megaspores near the micropyle end degenerate rapidly, and only the megaspore at the chalazal end survives as the functional megaspore (FM). The FM undergoes three successive mitotic divisions to form a syncytium and then cellularization to form a mature female gametophyte (FG) with a multicellular structure. The FG comprises an egg cell, a central cell, two synergid cells, and three antipodal cells. ii, inner integument; oi, outer integument; ch, chalaza; mp, micropyle; ac, antipodal cell; cc, central cell; sc, synergid cell; ec, egg cell.

Female gametophyte development is indispensable for sexual reproduction and the alternation of generations in flowering plants. Unlike animals, most flowering plants do not differentiate into germline cells during embryogenesis. Instead, they differentiate from somatic cells within floral meristems at the reproductive stage. In *Arabidopsis thaliana*, female germline development is a complex and precisely regulated biological process that comprises two successive stages: megasporogenesis and megagametogenesis [1,2]. Megasporogenesis initiates from a subepidermal layer (L2) nucellar cell at the distal end of the ovule primordium, which differentiates into the archesporial cell (AC) and subsequently into the megaspore mother cell (MMC). Subsequently, the MMC undergoes meiosis to form four haploid megaspores, three of which near the micropyle rapidly degenerate, whereas only the megaspore at the chalazal end survives and differentiates into the functional megaspore (FM). The FM undergoes three successive rounds of mitosis to generate a syncytium, followed by cellularization, eventually forming the mature female gametophyte (FG), namely the embryo sac, which consists of an egg cell, a central cell, two synergid cells and three antipodal cells [1] (Figure 1). Within each ovule, only one subepidermal somatic cell acquires MMC identity, and any disruption of this process directly compromises fertilization and seed production. Although numerous regulators of MMC specification have been identified, including epigenetic modifications, microRNA-mediated silencing [3,4,5,6,7,8], and phytohormone regulation [9,10,11,12,13,14], these studies have focused on individual pathways, and how multiple signals are integrated to differentiate a single MMC within each ovule remains poorly understood.

In *Arabidopsis thaliana*, the position of the female germline precursor is strictly constrained. During the formation of the ovule primordium, a cell either undergoes mitotic division as a somatic cell or differentiates into the MMC and enters meiosis [15], indicating that MMC specification is an intricate process governed by positional signaling cues. Following the classical framework of positional information and its recent conceptual refinements [16,17], we use the term “positional signals” to refer to mobile, spatially distributed cues that convey directional information among cells. Candidate positional signals include phytohormone gradients (e.g., brassinosteroids (BRs), auxin [18,19]), mobile small RNAs [7,20,21], and KLUH (KLU/CYP78A5)-dependent non-cell-autonomous signaling [22,23]. We reserve the complementary term “positional competence” for the cell-intrinsic machinery that perceives, transduces, and interprets these signals, encompassing chromatin states (e.g., H1/H3 eviction, H2A.Z incorporation [22,24,25,26,27,28]), physical barriers and transport pathways (e.g., callose deposition at the cell wall, cytoskeleton-driven directional transport [29,30]), and mechanical forces [31]. Positional signals define “where” information is delivered, whereas positional competence determines “which” cells are capable of responding and “how” the response is executed.

Building on this framework, we systematically summarize the current understanding of phytohormone gradients and mobile signals that act as candidate positional signals during MMC specification, and then discuss intrinsic mechanisms, including receptor positioning, cell wall-mediated physical barriers, cytoskeleton-driven directional transport, and mechanical force transduction, by which receiving cells interpret these signals to restrict MMC identity to a single somatic cell.

## 2. Key Regulators of MMC Specification

**Figure 2:**
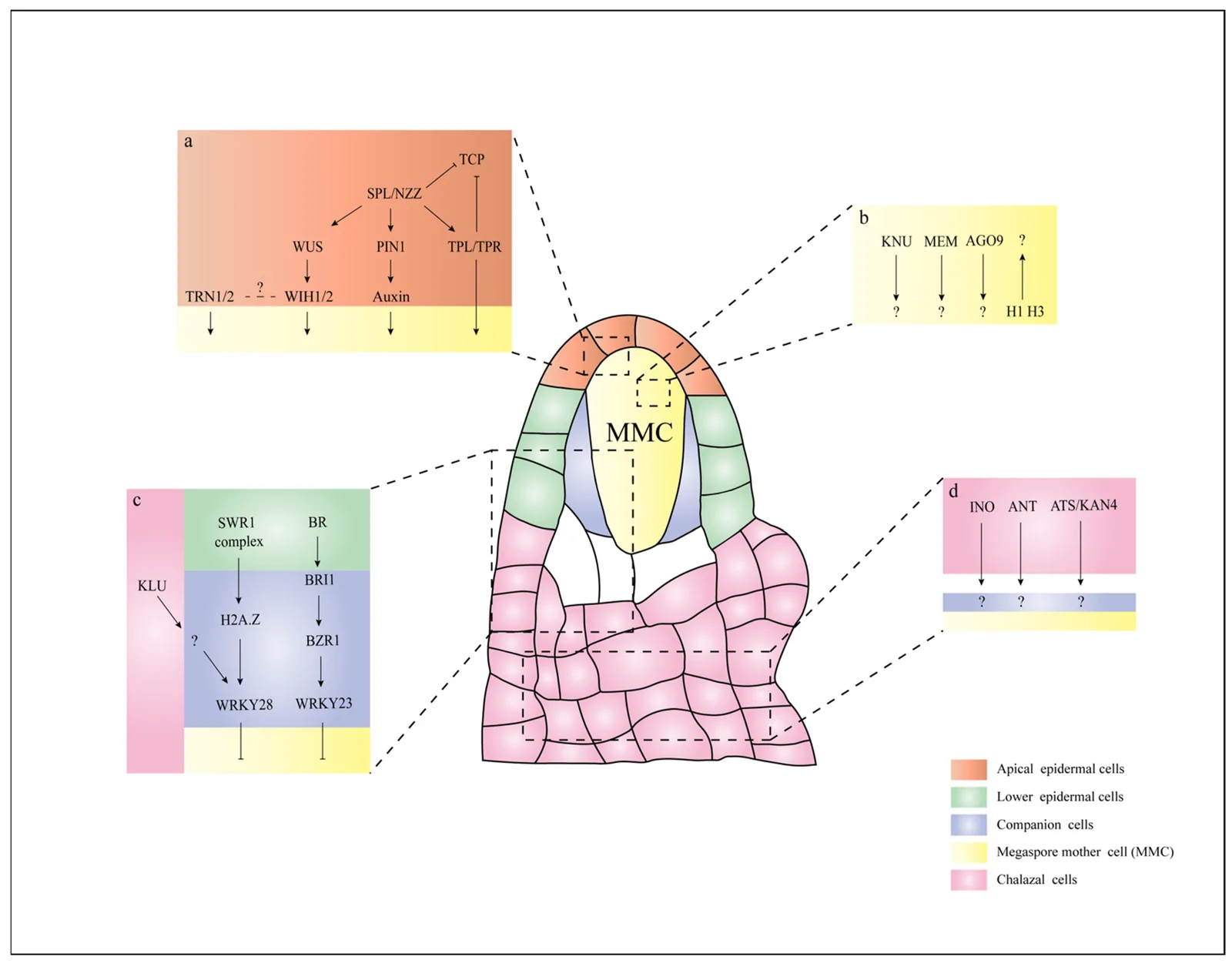
Key factors and signaling pathways regulating MMC specification in *Arabidopsis*. The major signaling pathways are shown in the apical epidermal cells (**a**), the MMC (**b**), companion cells (**c**), and chalazal cells (**d**). Question marks indicate uncharacterized upstream factors and downstream regulatory components that remain to be identified.

Along the distal-proximal axis, the early ovule primordium is spatially divided into three distinct domains: the distal nucellus, the intermediate chalaza, and the proximal funiculus. The nucellus occupies the most distal position and acts as the site of female germline initiation. Proximal to the nucellus is the chalaza (the region where the integuments adjoin the nucellus), which connects to the funiculus, linking the ovule to the placenta on the ovary wall. The integuments originate from the chalaza base of the ovule primordium [32,33]. During early ovule development, the cells of the outermost layer of the nucellus are defined as epidermal cells (also known as L1 layer cells), whereas the cells located below the epidermal cell layer of the nucellus are referred to as subepidermal layer cells (also known as L2 layer cells), which comprise the MMC and its companion cells (Figure 2a–d).

Beyond this spatial framework, numerous genes expressed in the L1 layer have been implicated in MMC development [15]. *SPOROCYTELESS/NOZZLE* (*SPL/NZZ*) is expressed in the L1 layer, and *spl/nzz* mutants exhibit no MMC formation [34], emphasizing the essential role of SPL/NZZ in MMC specification. SPL/NZZ recruits the co-repressor TOPLESS/TOPLESS-RELATED (TPL/TPR) via its C-terminal EAR domain to repress CINCINNATA (CIN)-like TEOSINTE BRANCHED1/CYCLOIDEA/PCF (TCP) transcription factors [35,36]. Moreover, aborted ovule phenotypes similar to those of *spl/nzz* are observed in *tpl* mutants and overexpression lines of TCP [36], indicating that these factors may belong to the same signaling pathway jointly regulating MMC formation. Similarly, the reduced expression of *WUSCHEL* (*WUS*) in *spl/nzz* mutants, together with the absence of the MMC in *wus* mutants, indicates that *WUS* acts downstream of *SPL/NZZ*. These results suggest that the transcription factor *WUS* and *SPL/NZZ* may act in the same genetic pathway to regulate MMC formation. In addition, WUS indirectly activates two redundant genes, *WINDHOSE1* (*WIH1*) and *WINDHOSE2* (*WIH2*). Although MMC formation is not affected in *wih1* and *wih2* single mutants, *wih1/2* double mutants exhibit no MMC [37]. Notably, the leaf-curling phenotype of *wih1/2* double mutants resembles that of *tornado1* (*trn1*) and *tornado2* (*trn2*) single mutants, suggesting that *WIH1*, *WIH2*, *TRN1* and *TRN2* are involved in the same signaling pathway regulating MMC formation. However, TRN1 is a cytoplasmic protein and TRN2 is a multi-transmembrane protein; it is unlikely that either acts as a direct receptor of WIH1 and WIH2 [1,37]. The mechanism by which WIH1/2 and TRN1/2 contribute to MMC formation remains unclear. Furthermore, a local auxin accumulation maximum occurs at the apical epidermal cell layer, which depends on its transporter PIN-FORMED (PIN1) [18,19]. This suggests a potential interaction between PIN1 and SPL/NZZ in the coordinated regulation of MMC development. Collectively, these results indicate that SPL/NZZ and WUS, expressed in L1 layer cells, establish an environment for MMC specification (Figure 2a).

Within the subepidermal cells that directly give rise to the MMC, only one somatic cell differentiates into the reproductive cell. Initially, the apical region of the ovule primordium extends into a finger-like structure and differentiates into an archesporial cell (AC), which subsequently differentiates into the MMC. Once the MMC starts to expand, its epigenetic and transcriptional states undergo rapid remodeling compared with those of surrounding sporophytic somatic cells. These changes involve condensed chromatin or heterochromatin dynamics, crucial histone modifications, and changes in core histone variants [24,38,39]. Recent studies using non-denaturing whole-mount staining and confocal imaging have revealed that MMC specification is accompanied by chromatin decondensation and a reduction in heterochromatin [25]. In addition, the linker histone (H1) binds to both the linker DNA and the core nucleosome, which regulates chromatin compaction to stabilize the chromatin structure [40]. There are three *H1* genes in *Arabidopsis*, including *H1.1*, *H1.2*, and *H1.3* [41]. Compared with surrounding somatic cells, the expression levels of H1.1 and H1.2 rapidly decrease during early development of MMC and become undetectable at subsequent stages, indicating that the induction of H1 may be linked to the specification of a somatic cell into the MMC [1,24,25,26,42]. Further research has found that the citrullinase AGMATINE IMINO HYDROLASE (AIH) catalyzes the citrullination of H1.1 at the R57 site, reducing its chromatin-binding affinity and promoting its dissociation. After dissociation, H1.1 is ubiquitinated at the K89 site by the E3 ubiquitin ligase CULLIN4 (CUL4) and ultimately degraded by the proteasome, ensuring the smooth progression of late gametogenesis [26]. Similar to H1.1 and H1.2, the histone variant H3.1 is also undetectable during MMC specification [27]. Immunofluorescence assays show that the levels of histone H3 lysine 4 trimethylation (H3K4me3) and H3K27me3 are reduced in the MMC compared with surrounding somatic cells, indicating that a large number of genes have undergone transcriptional reprogramming during MMC specification [25]. An important question arising from these findings is whether the epigenetic changes described above act as a cause or a consequence of positional signal interpretation. Current evidence suggests that H1 eviction is more likely a downstream consequence of upstream positional signals than a spontaneous cell-autonomous event, as the AIH-mediated citrullination and CUL4-mediated ubiquitination of H1.1 are triggered by external signals; H1.1 and H1.2 rapidly decrease only in the MMC. Similarly, the eviction of H3.1 and the reduction in H3K4me3/H3K27me3 are spatially restricted to the MMC, further supporting the existence of upstream positional signals. Collectively, these epigenetic changes enhance chromatin accessibility and transcriptional reprogramming, thereby establishing the positional competence that enables the receiving cell to interpret positional signals (e.g., auxin). However, whether they also directly promote the transcription of signal perception components remains to be explored. Beyond epigenetic remodeling, MMC specification is precisely regulated by genes expressed both in the MMC and companion cells [1,15]. The C2H2 zinc-finger protein KNUCKLES (KNU) is commonly used as a marker to confirm the MMC identity. It is specifically expressed in the MMC and facilitates the initiation of MMC development [43], but how it regulates MMC specification remains unclear. Another MMC identity marker is ARGONAUTE9 (AGO9), which is expressed at the cell membranes of ovule epidermal cells and the MMC; *ago9* mutants present multiple MMC-like cells in ovules [7,44]. Additionally, MNEME (MEM) is also specifically expressed in the MMC, which encodes an ATP-dependent RNA helicase belonging to epigenetic regulatory factors. The *mem* mutants exhibit multiple MMCs [8], yet whether these cells all acquire MMC identity and how MEM restricts MMC formation to a single somatic cell remains unclear. In companion cells, the zinc-finger WRKY transcription factors WRKY28 and WRKY23 prevent surrounding somatic cells from acquiring MMC identity. *wrky28* mutants exhibit multiple MMC-like cells. In addition, the brassinosteroid (BR) signaling pathway promotes *WRKY23* expression, thereby inhibiting surrounding somatic cells from acquiring MMC identity [9]. Additionally, KLU promotes *WRKY28* transcription by facilitating the deposition of the histone variant H2A.Z at the *WRKY28* locus via the SWI2/SNF2-RELATED 1 (SWR1) chromatin remodeling complex. This suggests that the mobile signals generated by KLU diffuse into subepidermal nucellar cells to activate *WRKY28* expression, thereby preventing companion cells from acquiring MMC identity [22]. It is hypothesized that other mobile signals in companion cells may also activate WRKY28 transcription. These results suggest that WRKY28 and WRKY23 are specifically expressed in companion cells and act as regulators that mediate positional signals to inhibit companion cells from obtaining MMC identity, ensuring that only one somatic cell acquires MMC identity (Figure 2b,c).

Most genes expressed in the chalaza predominantly regulate integument development [1], for example, *INNER NO OUTER* (*INO*) [45], *AINTEGUMENTA* (*ANT*) [46] and *ABERRANT TESTA SHAPE/KANADI4* (*ATS/KAN4*) [47]. Given the spatial separation between the chalaza and the nucellar cells, the mechanism by which chalazal genes participate in regulating MMC specification is currently elusive. Consistent with this role, KLU, which is expressed in the inner integument, integrates BR signaling and epigenetic regulation to promote the expression of WRKY28 in a non-cell-autonomous manner [22,23]. (Figure 2c,d).

## 3. Phytohormone Gradients as Spatiotemporal Positional Signals

Phytohormones are fundamental regulators of various plant growth and developmental processes [3]. Here, we specifically focus on BRs [9], auxin [19], gibberellins (GAs), and cytokinins (CKs) as crucial positional signals regulating MMC specification [10,19].

### 3.1. BR Gradients as a Positional Signal for MMC Specification

As essential phytohormones, BRs play crucial roles in regulating diverse stages of plant growth and development, as well as in responding to external environmental changes. Recent studies have found that BR biosynthesis and signal transduction components are predominantly expressed in the sporophytic tissues of the ovule rather than in the MMC. This suggests that BRs establish the concentration gradient within the ovule that acts as a positional signal for regulating the initiation and development of the MMC [9,19,33]. Single-cell sequencing across different ovule developmental stages has revealed that the transcription factor BRASSINAZOLE-RESISTANT 1 (BZR1) acts as an important regulator of MMC development [32]. Ovules of BR-deficient mutants exhibit multiple MMC-like cells, all of which enter meiosis, suggesting that BR signaling pathways are involved in regulating MMC specification [9]. Further research proposes that BRs establish concentration gradients within the nucellus, with the highest concentration detected in the L1 layer cells. The BR receptor BRASSINOSTEROID INSENSITIVE 1 (BRI1), together with the BZR1 transcription factor family, activates WRKY23 in companion cells. The activation of WRKY23 inhibits companion cells from acquiring MMC identity, ensuring that only one MMC is generated in each ovule [9].

Furthermore, recent studies have found that EPIDERMAL PATTERNING FACTOR-like (EPFL) peptide ligands expressed in the L1 layer cells form the EPFL-ER signaling pathway with ERECTA family (ER) receptor kinases. This pathway activates the expression of *BRI1* and *BZR1*; BZR1 subsequently activates a nucleolar GTP-binding protein, NUCLEOSTEMIN-LIKE 1 (NSN1), which is expressed in sporophytic somatic cells but not in the MMC, thereby restricting other subepidermal somatic cells from differentiating into MMCs [48].

Additionally, BZR1 is activated by the chromatin remodeling complex SWR1 and the H3K4me3 histone methyltransferase SET DOMAIN GROUP 2 (SDG2). The SWR1-SDG2-ER signaling pathway facilitates the accumulation of BZR1 protein in ovule cells; BZR1 then directly binds to and activates miRNA processing factors HYPONASTIC LEAVES 1 (HYL1), DICER-LIKE 1 (DCL1), and SERRATE (SE) to regulate MMC development [28].

**Figure 3:**
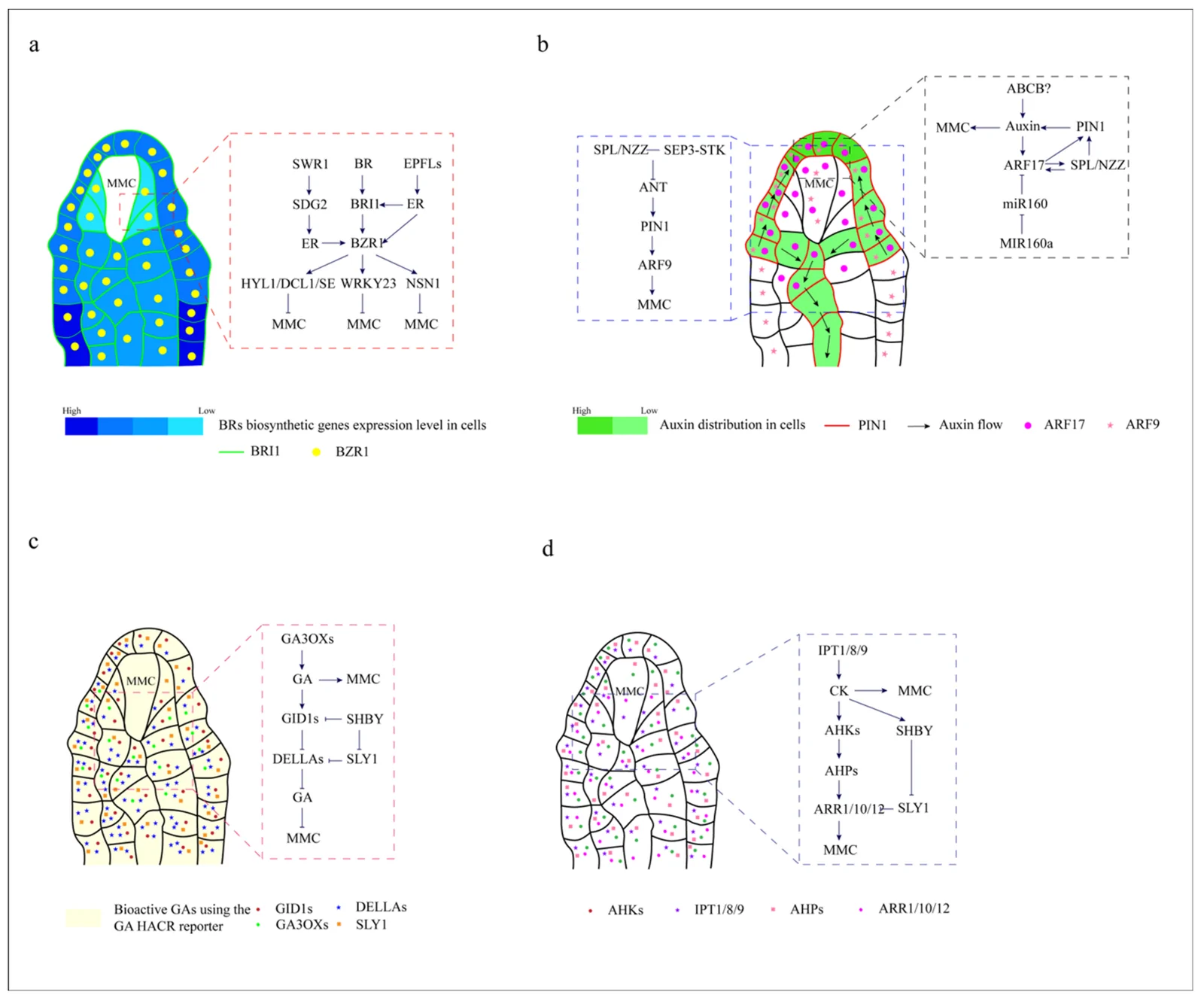
Phytohormone distribution and signaling networks underlying MMC specification in the ovule primordium. (**a**) Distribution of BRs. The color coding illustrates the expression level of BR biosynthesis genes in cells. The BR signaling GRN functions via the BRs/BRI1-EPFLs/ER/SWR1/SDG2/ER-BZR1-WRKY23/NSN1/HYL1/DCL1/SE pathway. (**b**) Distribution of auxin. The color coding in the diagram indicates auxin distribution levels in cells. Two auxin signaling pathways are involved in MMC specification: the miR160-ARF17-SPL/NZZ-PIN1-auxin pathway, and the SPL/NZZ-ANT-PIN1-ARF9 pathway. The Question mark denotes a putative ABCB auxin transporter whose function remains to be verified. (**c**) Distribution of GAs. The color coding illustrates the distribution of bioactive GAs detected by the GA HACR reporter. The GA signaling GRN acts via the GA3OXs-GA-GID1s-DELLAs-GA pathway. (**d**) Distribution of CKs. The CK signaling GRN operates via the IPT1/8/9-CK-AHKs-AHPs-ARR1/10/12 pathway.

Collectively, these results illustrate that BRs and EPFL signals function as positional signals, whereas the localization of BRI1 and ER receptors, together with the BZR1-centered transcriptional and chromatin-level network, constitutes the positional competence machinery that interprets these signals to ensure that only one somatic cell acquires MMC identity (Figure 3a).

### 3.2. Auxin Gradients as a Positional Signal for MMC Specification

Auxin serves as an important regulatory factor in plant growth and development [14]. It provides essential positional cues for MMC development by establishing concentration gradients within the ovule. Unlike other phytohormone signals, auxin concentration gradients rely on local biosynthesis and polar intercellular transport, which are mediated by the auxin biosynthesis gene *YUCCA1* (*YUC1*) [18], the auxin efflux carrier PIN1, and the ATP-binding cassette sub-family B (ABCB) transporter [18,19,49]. When auxin accumulates in cells, AUXIN RESPONSE FACTORs (ARFs) are activated to promote the expression of auxin-responsive genes [50]. During early ovule development, the DR5rev::GFP reporter reveals auxin accumulation and concentration gradients in the L1 layer of ovules [18,50]. Ectopic expression of *YUC1*, driven by the *EMS1* promoter that is actively expressed in the nucellar and chalazal cells, results in the formation of multiple MMCs within ovules [18]. This suggests that the high concentration of auxin promotes MMC specification.

The *pin1* mutant and the application of the PIN1-mediated polar auxin transport inhibitor Naphthylphthalamic Acid (NPA) cause auxin diffusion from the epidermal layer into the entire ovule, thereby disrupting auxin concentration gradients in the L1 layer cells and resulting in the formation of multiple MMCs. These results indicate that PIN1 serves as a key transporter for polar auxin transport; its polar plasma membrane localization and dynamic expression in the nucellus establish the auxin concentration gradients at the epidermis of the ovule primordium, providing crucial positional signals for MMC specification [18,19,50,51].

Furthermore, miR160 and ARF17 regulate the expression of *PIN1* [18]. The *mir160* and *ARF17m* (*miR160*-resistant version) mutants exhibit PIN1 extension into the chalaza, altering auxin concentration gradients and leading to the formation of multiple MMCs, all of which obtain the molecular characteristics of the MMC [18]. Reduced expression levels of auxin-responsive genes *DR5* and *PIN1* were observed in *spl/nzz* mutants, indicating that SPL/NZZ plays an important role in the auxin signaling pathway [18,52]. SPL/NZZ genetically interacts with ARF17 to establish a single local auxin maximum at the apical epidermis. Both *mir160* and *ARF17m* mutants can restore MMC formation in *spl* mutants [18,19]. This finding suggests that the miR160-SPL/NZZ-PIN1 regulatory module is indispensable for establishing the maximum auxin concentration at the apical epidermal cells and thereby participates in MMC specification. Furthermore, SPL/NZZ interacts with the ovule identity transcription factors SEEDSTICK (STK) and SEPALLATA3 (SEP3). This transcriptional complex represses the expression of *ANT* at the chalaza, thereby preventing ectopic auxin accumulation in non-apical regions. At its normal expression domain, ANT promotes *PIN1* expression to establish the local maximum of auxin, which in turn activates *ARF9* expression to promote MMC specification [53]. Notably, ARF17 and ARF9 play distinct roles in signaling pathways: ARF17, together with miR160 and SPL/NZZ, acts upstream to regulate PIN1 expression and establish the auxin maximum, whereas ARF9 functions downstream, being activated by the auxin maximum to regulate MMC specification. However, the role of another auxin transporter, ABCB, in MMC specification is still unclear.

Collectively, these findings reveal that the auxin maximum at the apical epidermis functions as the positional signal, whereas the YUC1-mediated local biosynthesis, PIN1-dependent polar transport, and the miR160-SPL/NZZ-STK/SEP3 regulatory network constitute the positional competence machinery that generates and interprets this gradient to restrict MMC identity to only one somatic cell in each ovule (Figure 3b).

### 3.3. GA Distribution as a Putative Positional Signal for MMC Specification

GAs also play essential roles in various plant developmental processes, including seed germination, organ formation, flowering time and embryo sac development [33,54]. Several key components of GA signaling pathways have been identified during MMC specification, notably the GA receptor GA-INSENSITIVE DWARF1 (GID1) and DELLA proteins, which play important roles in regulating ovule initiation and the number of ovules [12,55,56].

Binding of bioactive GA induces a conformational change in the N-terminal extension of GID1, enabling GID1 to bind DELLA proteins and promote their degradation, thereby activating GA signaling [55]. DELLA proteins are members of the plant-specific GRAS family and act as GA-signaling repressors. The GA-GID1-DELLA complex triggers the rapid degradation of DELLA proteins, thereby activating GA signaling [57,58]. SLEEPY 1 (SLY1) is the F-box subunit of the Skp1-Cullin-F-box (SCF) E3 ubiquitin (Ub)-ligase complex, which interacts with DELLA substrates to promote their ubiquitination and degradation, thereby activating GA signaling [59]. In *Arabidopsis thaliana*, the GID1 family consists of three members: GID1a, GID1b, and GID1c. The fertility of *gid1a/b* and *gid1a/c* double mutants is significantly reduced; the fertility of *gid1b/c* double mutants remains unaffected, whereas the *gid1a/b/c* triple mutants are completely sterile. Further research has indicated that the complete sterility of *gid1a/b/c* mutants results from abnormal female gametophyte development, while MMC specification remains unaffected [56]. *Arabidopsis* has five DELLA proteins, namely GA-INSENSITIVE 1 (GAI), REPRESSOR OF GA (RGA), RGA-LIKE 1 (RGL1), RGL2, and RGL3. The *gai*, *rga*, *rgl1*, *rgl2*, and *rgl3* single mutants, as well as the della quintuple mutants (“*della-q*”; *gai rga rgl1 rgl2 rgl3*), exhibit multiple MMCs, which all obtain the molecular characteristics of MMCs but fail to enter meiosis [10,59]. Exogenous application of 100 μM bioactive GA_4+7_ to wild-type (WT) ovules induces multiple MMCs in about 17% of treated ovules. All of them acquire MMC molecular characteristics, while only one enters meiosis [10]. Recent studies have found that GIBBERELLIN 3-OXIDASEs (GA3OXs) [60], which catalyze the biosynthesis of GA, as well as *GID1s*, *DELLAs* and *SLY1*, are predominantly expressed in the sporophytic tissues rather than in the MMC [10,19]. Overexpression of *GID1s* or *GA3OX4*, driven either by the ovule-specific *SEEDSTICK* (*STK*) promoter or by the constitutive *CaMV35S* promoter, results in the formation of multiple MMC-like cells within the ovule. Among these, only one is confirmed as the MMC, while the remaining enlarged cells undergo developmental arrest and fail to enter meiosis [19,33,61]. Moreover, the use of a GA-detecting sensor (GA HACR, hormone-activated Cas9-based repressor) has revealed that endogenous bioactive GA accumulates in the MMC and surrounding somatic cells, underscoring the importance of endogenous GA in the specification of MMC [19].

These results demonstrate that MMC specification requires the suppression of GA signaling; the spatial distribution of bioactive GA within the ovule constitutes a putative positional signal, while the GA biosynthetic enzymes, receptors, and downstream signaling components constitute the positional competence machinery that ensures that only one somatic cell acquires MMC identity in each ovule (Figure 3c).

### 3.4. CK Distribution as a Putative Positional Signal for MMC Specification

CKs are integral to various stages of plant growth and development, significantly influencing plant cell division and differentiation [62]. In *Arabidopsis thaliana*, six CK receptors have been identified, namely ARABIDOPSIS HISTIDINE KINASES 2 (AHK2), AHK3, AHK4/CRE1/WOL, AtHK1, CKI1, and CKI2/AHK5. Among these, AHK2, AHK3, and CRE1 exhibit highly conserved amino acid sequences, particularly in the extracellular CK-binding domain. In addition, they are widely distributed during ovule development [52,63]. The *ahk2-2 ahk3-3 cre1-12* triple mutants maintain a long finger-like structure in the ovule primordium, thereby preventing the ovule from entering female gametophyte development [52].

Upon CK binding, AHK2-4 undergo autophosphorylation and transfer the phosphate group to histidine phosphotransfer proteins AHPs, which comprise five members (AHP1-5) in *Arabidopsis*. AHPs can also be phosphorylated by the histidine kinase CYTOKININ INDEPENDENT 1 (CKI1), which lacks CK-binding activity. AHPs subsequently transmit phosphorylation signals to *Arabidopsis* response regulators (ARRs), which control the transcription of CK-responsive genes [64]. ARRs are classified into two types: type-B ARRs, which function as transcriptional activators, and type-A ARRs, which are involved in negative feedback. The type-B *arr10/12/18*, *cki1*, and *ahp2/3/5* mutants lose central cell identity in the ovule primordium, while constitutively active ARR10/12/18 partially rescues the central cell defects in *cki1* and *ahp2/3/5* mutants [65]. These findings suggest that the CKI1-AHP2/3/5-ARR10/12/18 signaling module plays an important role in female gametophyte development. The CK synthesis gene *IPT* (*isopentenyltransferase*) is expressed throughout all stages of ovule development in *Arabidopsis*, and *ipt* mutants form multiple MMCs [10,19,66]. These results demonstrate that the loss or disruption of CK signaling leads to the formation of multiple MMCs, emphasizing the critical role of CK signaling in MMC specification. The expression patterns of AHKs, AHPs, and ARR1/10/12 are similar to those of IPT, which is expressed in both sporophytic tissues and the MMC. Further research has found that the specific activation of ARR1, driven by either the companion cell-specific *WRKY28* promoter or the MMC-specific *KNU* promoter, completely rescues the MMC defects in *arr1/10/12* mutants. This suggests that ARRs can simultaneously regulate the identities of both the MMC and companion cells [10].

Consistent with the framework established for BRs, auxin, and GAs, the spatial distribution of CKs throughout the ovule functions as a positional signal. Meanwhile, the CK perception and transduction machinery, including AHK receptors, AHPs, and ARR transcription factors, constitutes the positional competence that enables receiving cells to interpret CK signals and execute the appropriate cell identity decision. Therefore, CK signaling likely acts as a positional signal to ensure that only one somatic cell in the nucellus acquires the MMC identity, while the AHKs-AHPs-ARR1/10/12 module serves as the positional competence machinery that interprets this signal (Figure 3d).

### 3.5. Crosstalk Among Phytohormone Positional Signals in MMC Specification

Phytohormones rarely act in isolation; they form interconnected networks that coordinately regulate MMC specification. As a positional signal, auxin exhibits the highest concentration in the L1 layer, promoting the specification of one somatic cell into the MMC. In contrast, CKs are distributed throughout sporophytic somatic cells, preventing companion cells from acquiring MMC identity. These two signals establish a distal-proximal concentration gradient that regulates MMC specification [67].

Beyond auxin-CK interactions, recent studies have also revealed crosstalk between GA and CK pathways. The vacuolar sorting protein SHRUBBY (SHBY) functions as a key integrator of GA and CK positional signaling. SHBY promotes the ubiquitination and degradation of GID1 and SLY1, thereby maintaining the stability of both DELLA and ARR proteins. GID1 directly interacts with ARR1/10/12 and recruits the SLY1 E3 ubiquitin ligase to these ARRs, forming the GID1-SLY1-ARRs complex. This complex promotes the ubiquitination and degradation of ARRs, ultimately suppressing CK signaling. Conversely, CKs enhance the stability of DELLA proteins through SHBY to repress GA signaling, while GAs promote ARR protein stability through SHBY to activate CK signaling. GAs and CKs precisely co-regulate MMC specification by dynamically balancing the stability of their downstream positional competence components [10] (Figure 3b–d).

These results demonstrate that the gradient distribution of phytohormones within the ovule serves as a positional signal, whereas downstream signaling components constitute the positional competence machinery that restricts MMC identity to a single somatic cell within each ovule.

## 4. Mobile Signals as Positional Signals in MMC Specification

Apart from phytohormones, mobile signals serve as important carriers of positional information. In this review, we restrict the discussion to mobile signals with experimental evidence in ovule development, including mobile small RNAs and KLU-dependent signals. Their intercellular trafficking through plasmodesmata enables them to act as positional signals for MMC specification. Candidate signals lacking direct evidence in ovule development (e.g., ROS and Ca^2+^) are discussed in Section 5.

### 4.1. Mobile Small RNAs as Positional Signals for MMC Specification

Small RNAs act as mobile silencing signals that are crucial for MMC specification. Mutations in *ARGONAUTE 9* (*AGO9*), which binds to 24-nt small RNAs (sRNAs) derived from transposable elements (TEs) and silences these TEs, result in the presence of multiple MMCs. Similarly, mutations in *RNA-DEPENDENT RNA POLYMERASE 6* (*RDR6*) and *SUPPRESSOR OF GENE SILENCING 3* (*SGS3*), which are involved in siRNA biosynthesis, as well as in RNA-directed DNA methylation (RdDM) silencing components, also lead to multiple MMCs [7,20,21]. The *rdr6* and *sgs3* single mutants exhibit excessive MMCs in the ovule, a phenotype similar to that of *ago9* mutants [7]. These findings suggest that the outward movement of sRNA silencing signals from companion somatic cells is essential for restricting MMC identity to a single somatic cell.

Beyond general siRNA-mediated silencing, a specific class of mobile TAS3-derived trans-acting small interfering RNAs (ta-siRNAs) has also been implicated in MMC specification. TEX1, HPR1, and THO6 are important components of the THO/TREX complex. *tex1*, *hpr1* and *tho6* single mutants all exhibit multiple MMCs in ovules. Further investigation has found that TEX1 is distributed in the L1 layer of ovules, together with RDR6, and promotes the biosynthesis of ta-siRNAs. These ta-siRNAs restrict AUXIN RESPONSE FACTOR 3 (ARF3) to the chalaza and inhibit its expression. In the *tex1* mutant, the expression domain of ARF3 expands from the chalaza to the L1 layer of ovules, resulting in the formation of multiple MMCs [1,68,69]. Specifically, epidermal ta-siRNAs prevent excessive somatic cells in the L2 layer from acquiring the identity of MMC by restricting ARF3 to the chalaza and inhibiting its expression.

**Figure 4:**
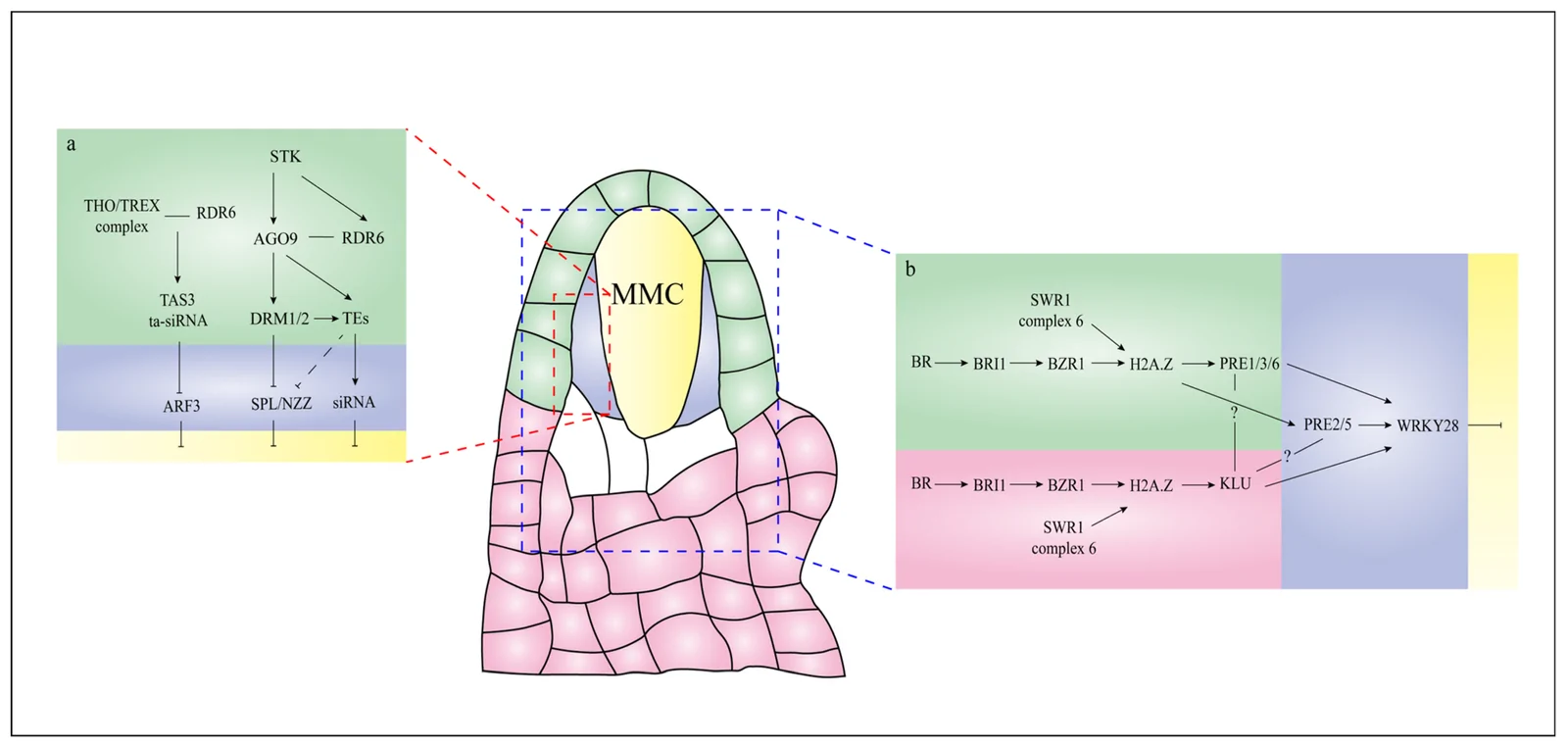
Mobile positional signals in MMC specification. (**a**) Mobile small RNAs: siRNAs from companion cells act non-cell-autonomously to restrict MMC identity. (**b**) KLU-dependent signaling: KLU-dependent mobile signals involved in MMC specification. Question marks indicate unknow factors or mechanisms that remains to be identified.

In addition to ta-siRNA-mediated regulation, the small RNA pathway also regulates gene expression through AGO-mediated mRNA degradation and through RNA-directed DNA methylation (RdDM), whereby cytosine methylation of target genes is catalyzed by DOMAINS REARRANGED METHYLTRANSFERASES 1 AND 2 (DRM1/DRM2). *drm1/2* mutants exhibit supernumerary MMCs, a phenotype similar to that of *stk*, *ago9*, and *rbr6* single mutants [6], of which only one acquires MMC identity. The *stk*, *ago9*, *rdr6,* and *drm1/2* mutants all display ectopic expression of *SPL/NZZ*, suggesting that the formation of excessive MMC-like cells may be due to the ectopic activation of SPL/NZZ. Further research shows that the STK directly regulates the expression of *AGO9* and *RDR6*, and AGO9 in turn regulates *DRM1/2* and *SPL/NZZ* expression, thereby ensuring that only one cell in the ovule acquires MMC identity [6] (Figure 4a).

### 4.2. KLU-Derived Mobile Signals as a Putative Positional Signal for MMC Specification

The cytochrome P450 gene *KLU* generates signaling molecules involved in leaf and flower organ development in a non-cell-autonomous manner [22,70,71]. Additionally, *KLU* is expressed in the inner integument. It is involved in regulating female meiosis and controlling seed size [22,72,73]. Moreover, KLU non-cell-autonomously promotes the deposition of the histone variant H2A.Z at the *WRKY28* locus via the SWR1 chromatin-remodeling complex to activate its expression, thereby preventing companion somatic cells from acquiring MMC identity [22].

Recent studies have identified the PRE family of transcription factors, including PRE1, PRE2, PRE3, PRE5, and PRE6, as key regulators in this process. They are mainly expressed in the distal nucellus region, while KLU is expressed in the proximal chalaza region of the ovule primordium, showing a spatially complementary expression pattern. Further research has demonstrated that KLU restricts PREs from diffusing into the chalaza region by interacting with the M8 motif of PREs, thereby limiting PREs to specific regions during MMC differentiation. The transcription factor BZR1, a component of the BR signaling pathway, directly activates the expression of *KLU* and *PREs*. BZR1 interacts with SWC6, a subunit of the SWR1 complex. This interaction promotes H2A.Z deposition at PREs loci and affects the chromatin state, ultimately activating downstream *WRKY28* expression [23].

Together, these results define a KLU-PREs module establishing spatial positional signals. Within this module, KLU confines PREs to the specific region and integrates BR signaling with SWR1-mediated chromatin remodeling to activate the expression of *WRKY28*. This process prevents companion cells from obtaining MMC identity, ensuring the correct initiation of the female germline (Figure 4b).

Consistent with the framework established for phytohormones, the mobile small RNAs and KLU-derived signals act as positional signals, while the downstream silencing machinery and the KLU-PREs-BZR1-SWR1-WRKY28 cascade constitute the positional competence machinery that interprets these signals to ensure that only one somatic cell acquires MMC identity.

## 5. Physical Regulation of Positional Signal Flow by the Cell Wall and the Cytoskeleton

In contrast to animals, female reproductive cells in plants differentiate from somatic cells. This process depends not only on intrinsic gene expression but also on positional signals. Positional signals pass through and are interpreted in a physical cellular framework. Within this framework, the cell wall and the cytoskeleton act as the major physical regulators of MMC specification. The cell wall gates intercellular signal movement through plasmodesmata, whereas the cytoskeleton provides tracks for chemical gradients, patterns division planes, regulates plasmodesmata permeability together with the cell wall, and transduces mechanical force.

### 5.1. Cell Wall Remodeling Gates Intercellular Signal Movement

Cell wall synthesis and modification enzymes, including CESAs, CSLs, GSL/CALS, and arabinogalactan proteins (AGPs), a class of cell wall proteins, show differences across different developmental stages of female germ cells. This indicates that cell wall components, cellulose, callose, and arabinogalactans play important roles in female reproductive cell differentiation [29]. Callose deposition is essential for MMC specification. By establishing a physical barrier around the MMC, it blocks plasmodesmata and severs symplastic connectivity between the MMC and surrounding somatic cells, thereby preventing mobile signals (such as small RNAs) from entering the MMC [29]. Furthermore, transcripts of *AGP18* can be transferred from the integument to the germ cells, influencing the formation of the functional megaspore (FM) [74]. As somatic cells transform into reproductive cells, their transcriptional and epigenetic states undergo rapid remodeling [24,38,39]. Importantly, this cellular transformation simultaneously occurs with cell wall remodeling [29], but whether cell wall dynamics act upstream of this reprogramming remains elusive. Collectively, the cell wall likely acts as a dynamic boundary that is actively remodeled during female germline specification.

**Figure 5:**
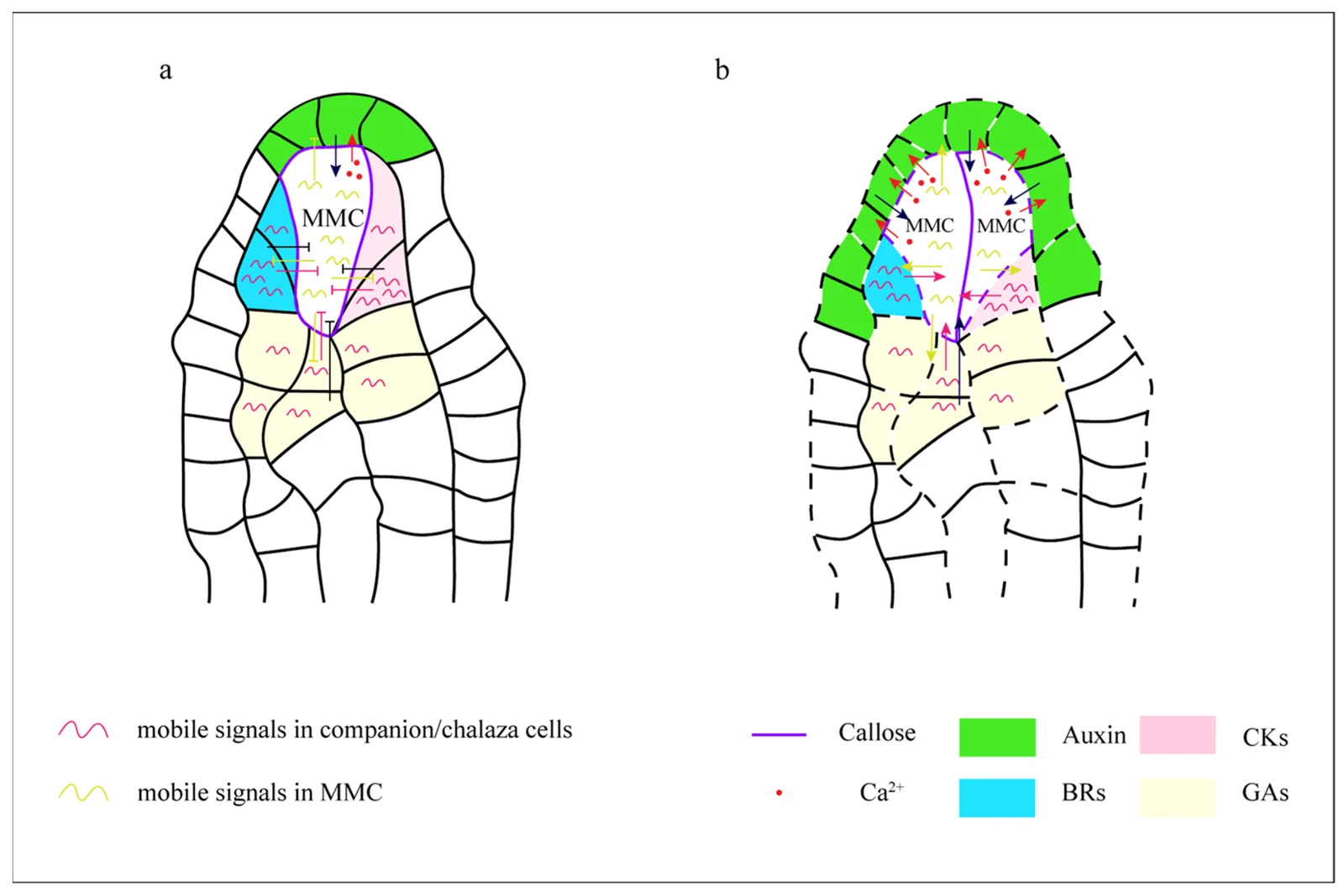
Model of phytohormone distribution and physical regulation during MMC specification in *Arabidopsis* ovules. (**a**) Cytoskeleton-mediated polar localization of PIN1 in nucellar cells establishes the epidermal auxin maximum. BRs and CKs distribute in companion cells, whereas GAs distribute in chalaza cells. These phytohormones are proposed to spatially antagonize auxin and then define the reproductive domain for MMC specification. Dynamic cell wall modifications and actin-regulated plasmodesmata gating establish the signaling isolation zone. Callose deposition modulates plasmodesmata permeability, which prevents intercellular trafficking of inhibitory signals from somatic cells and activating signals from the MMC. Cells positioned at the auxin maximum with the distinct cytoskeletal configuration generate mechanical forces to induce Ca^2+^ influx, initiate a phosphorylation cascade to activate SPL/NZZ, and specify a single somatic cell as the MMC via asymmetric division. (**b**) Latrunculin B-mediated actin depolymerization alters plasmodesmata permeability, resulting in widespread Ca^2+^ movement, aberrant signal exchange between the MMC and somatic cells, and disturbed auxin distribution, ultimately leading to the formation of multiple MMCs. The arrow indicates that mobile signals can freely enter other cells to facilitate intercellular communication; the T-bar arrow indicates that mobile signals cannot freely enter other cells. Color-coded arrows represent mobile signals of corresponding colors that mediate intercellular communication.

The remodeling of cell wall components, especially callose and AGPs, alters plasmodesmata permeability and mediates signal transmission by restricting or permitting intercellular signal movement to ensure that only one somatic cell acquires the MMC identity (Figure 5a).

### 5.2. Cytoskeleton-Mediated Auxin Gradient Formation and Division Plane Patterning

The cytoskeleton is composed of microtubules and microfilaments. It acts not merely as a structural scaffold for cell morphology, but also as an active executor and regulator of positional signaling networks. The skeleton establishes tracks for chemical gradients [75], mediates intercellular signal transmission [76], determines the cell division plane [30,77,78], and transduces mechanical signals [31]. Although it is currently difficult to directly identify cytoskeletal proteins that are specifically and transiently expressed during MMC specification, various conserved cytoskeletal regulators play important roles in the specification and functional maintenance of the MMC.

Polar auxin transport is a key positional signal for MMC specification, a process that depends on the polar localization of auxin transporters [18,19]. The polar localization of PIN1 relies on actin cytoskeleton-mediated vesicle trafficking. In mutants of Rab GTPase-modifying enzymes (such as *rgtb1*), the polar localization of PIN1 and PIN3 becomes disorganized. This disorganization reduces auxin efflux from the ovule, leading to abnormal accumulation of auxin in the tissues surrounding the developing female gametophyte. Consequently, mutant ovule development arrests at the mononuclear or binuclear stage, accompanied by abnormal integument development [75]. Furthermore, actin regulates nuclear positioning, thereby determining the location of the egg cell within the embryo sac [79].

The plant cell division plane depends heavily on the dynamic reorganization of microtubules and their associated proteins. The preprophase band (PPB) and the mitotic spindle, which are composed of microtubules, precisely determine the division plane position. The PPB marks the site of future cell wall formation, ensuring that two daughter cells differ in size and position [30]. The MMC is typically specified through the asymmetric division of a subepidermal nucellar cell. It is proposed that the microtubules comprising the PPB participate in MMC specification by marking the division plane of the subepidermal nucellar cell. Consequently, the larger apical daughter cell differentiates into the MMC, while the smaller basal cell maintains somatic cell identity. The γ-tubulin complex protein 4 (GCP4), a component of the microtubule nucleation complex, plays a critical role in microtubule (MT) nucleation. It influences the nucleation angle of cortical microtubules, spindle formation, and chromosome segregation. *gcp4* mutants exhibit abnormal female gametophyte development, ultimately resulting in aborted ovules and seed sterility. GCP4 may affect spindle orientation, resulting in the formation of two similarly sized daughter cells, thereby disrupting MMC differentiation [77,78]. In addition, the microtubule-severing protein KATANIN1 (KTN1) is crucial for cell division and cell morphogenesis. *ktn1* mutants display abnormal ovule development and embryonic cell division, resulting in twin embryos and seed sterility [80,81]. While current research primarily focuses on the role of KTN1 during late stages of female gametophyte development, its biological functions suggest a possible role in the asymmetric division underlying MMC specification. Collectively, these results indicate that the cytoskeleton establishes the physical framework for auxin gradient formation and patterns the division plane during MMC specification.

### 5.3. Actin Microfilaments Regulate Plasmodesmata Permeability for MMC Specification

In addition to providing tracks for vesicle trafficking, the microfilament network acts as a gatekeeper for positional signals by regulating plasmodesmata permeability. Actin microfilaments directly bind to the neck region of plasmodesmata and dynamically adjust the size exclusion limit (SEL) through contraction or dilation. Research has found that molecules up to 20 kDa can move intercellularly within the female gametophyte, and this connectivity strictly depends on intact microfilament skeletons. Treatment with Latrunculin B, which induces microfilament depolymerization and alters plasmodesmata permeability, disrupts the transmission of inhibitory signals among somatic cells [76], resulting in the simultaneous differentiation of multiple somatic cells into MMCs. During the MMC specification window, dynamic microfilament rearrangement may selectively close some channels between the future MMC and surrounding somatic cells, preventing inhibitory signals (like AGO9-mediated small RNAs) from entering the future MMC. Alternatively, they may open specific channels to permit activating signals to enter the future MMC (Figure 5a,b).

### 5.4. Cytoskeleton-Mediated Mechanical Signal Transduction for MMC Specification

The cytoskeleton also acts as a key sensor of the physical environment (including cell shape and tissue tension). It constitutes a potential component of positional competence by transducing the mechanical environment of the MMC. The MMC is generally larger than surrounding somatic cells and possesses a distinct aspect ratio. This morphology is maintained by cytoskeleton-directed cell wall deposition and turgor pressure. Cortical microtubules and microfilaments sense mechanical stress distribution across the cell wall and trigger Ca^2+^ influx through mechanosensitive ion channels (such as the MSL and MCA families), thereby altering intracellular auxin concentration gradients [31]. As a second messenger, Ca^2+^ activates calcium-dependent protein kinases (CDPKs), which phosphorylate downstream transcription factors or chromatin-modifying enzymes [31]. Whether these pathways operate during MMC specification, for example, through phosphorylation of MMC key determinants such as SPL/NZZ, or through regulation of intracellular auxin concentration gradients, remains to be demonstrated (Figure 5a).

Mechanical stress also induces the assembly of cytoplasmic stress granules (SGs) and processing bodies (P-bodies), the latter being primary locations for miRNA-induced silencing complex (miRISC) activity [82,83,84]. SGs protect mRNAs from degradation, whereas P-bodies mediate miRNA-directed decay or repression. If such assemblies form during MMC specification, SGs could stabilize transcripts encoding MMC determinants while P-bodies restrict the expression of competing MMC identity regulators (Figure 5a).

Collectively, current evidence supports the view that a somatic cell located at a distinct position, subjected to specific mechanical pressure, and possessing an intact cytoskeletal network is primed to differentiate into the MMC. Given the lack of direct evidence, we present this dimension as a testable hypothesis within the positional competence framework rather than as an established mechanism.

## 6. Conclusions and Future Perspectives

The MMC serves as the initial female germ cell in most flowering plants. Therefore, it is essential to elucidate the molecular mechanisms underlying MMC specification. In this review, we summarize the roles and of phytohormone gradients, mobile signals, cell wall physical barriers, and the cytoskeleton during MMC specification from the perspective of positional signals and positional competence. A central conclusion is that MMC specification is not the linear result of any single signaling pathway; rather, it emerges from the intricate coupling of four interconnected modules: chemical gradients established by phytohormones, intercellular communication mediated by mobile signals, physical barriers imposed by the cell wall, and mechanical perception mediated by the cytoskeleton.

Phytohormone gradients shape the chemical microenvironment for cell identity determination: auxin forms a local concentration maximum at the apical epidermis of the nucellus, which aligns with the expression region of SPL/NZZ and regulates MMC specification [18,19,52]. Biosynthetic enzymes and signaling regulators for BRs and GAs are expressed in nucellar somatic cells rather than in the MMC [9,10,12,19,28,48], whereas CK biosynthesis and regulatory factors are expressed in both nucellar somatic cells and the MMC [10,33,66]. These factors are proposed to antagonize the apical auxin signal, ensuring that only one somatic cell acquires MMC identity. AGO9, expressed in somatic cells surrounding the MMC, is an AGO9-dependent mobile siRNA that inhibits companion cells from acquiring MMC identity through plasmodesmata [6]. KLU-dependent non-cell-autonomous signaling integrates BRs and epigenetic mechanisms to prevent somatic cells around MMC from differentiating into MMCs [22,23]. Together, these mobile and physical barriers restrict surrounding somatic cells from obtaining MMC identity through plasmodesmata. Furthermore, callose deposition alters plasmodesmata permeability, acting as a dynamic filter for both inhibitory and activating signals. Beyond these chemical and physical barriers, the cytoskeleton integrates diverse signals: microtubules influence the polar localization of PIN1, thereby altering auxin concentration gradients [75], and determine the asymmetric division plane via the preprophase band and the spindle [30]. Microfilaments affect intercellular signal communication by controlling the pore size of plasmodesmata [76]. In addition, it is hypothesized that the cytoskeleton transforms the mechanical tension of the MMC into biochemical signals, such as Ca^2+^ influx, or assembles SGs and P-bodies in the cytoplasm to assist one somatic cell in differentiating into the MMC.

Based on recent studies, we propose a multidimensional positional signal integration model for MMC specification in *Arabidopsis*. Nucellar cells establish an epidermal auxin maximum through cytoskeleton-mediated polar localization of PIN1. Simultaneously, auxin, together with BRs, GAs, and CKs, defines the potential reproductive region through spatial antagonism. Within this microenvironment, cell wall undergoes dynamic modifications; together with microfilament-mediated gating of plasmodesmata, form a local signal isolation zone, while mobile signals generated by surrounding somatic cells establish an inhibitory field. A cell positioned at the auxin maximum and possessing the specific cytoskeletal configuration can overcome this inhibition and then specifically activate SPL/NZZ. Subsequently, through asymmetric division, only one somatic cell acquires the MMC identity (Figure 5a,b).

Although this framework has been initially established, several questions remain unsolved. Firstly, while phytohormone concentration gradients function as positional signals during MMC specification, it is uncertain whether the distribution of other phytohormones within ovules also depends on the cytoskeleton, and what the underlying molecular mechanisms might be. Secondly, the BR receptor BRI1 exhibits a polar distribution in ovules [9]. Does this polar distribution determine BR distribution in ovules, similar to the relationship between the auxin transporter PIN1 and auxin, and does it also rely on the cytoskeleton? Thirdly, in addition to trafficking through plasmodesmata, it remains uncertain whether intercellular mobile signals transmit via other cytoskeletal proteins, such as motor proteins. Finally, how are mechanically sensitive ion channels (such as the MSL and MCA families) activated? Upon activation, apart from triggering Ca^2+^ influx, what other signals might be triggered? What are molecular mechanisms by which these signals regulate MMC specification?

Future research should further identify specific cytoskeletal proteins and mechanically sensitive channels to fully elucidate signaling pathways of this complex network. This understanding also provides new theoretical targets for crop genetic improvement.

## Data Availability

This is a review article, and no new experimental data were generated. All data discussed in this review are available in the cited published literature.
